# Investigating the Prognostic Significance of Pyroptosis-Related Genes in Gastric Cancer and Their Impact on Cells’ Biological Functions

**DOI:** 10.3389/fonc.2022.861284

**Published:** 2022-03-28

**Authors:** Jie Yin, Gang Che, Wankun Wang, Shitu Chen, Jian Liu

**Affiliations:** ^1^ Department of Radiation Oncology, The First Affiliated Hospital, Zhejiang University School of Medicine, Hangzhou, China; ^2^ Department of Surgical Oncology, The First Affiliated Hospital, Zhejiang University School of Medicine, Hangzhou, China

**Keywords:** gastric cancer, pyroptosis, GSDME, cell proliferation, pyroptosis-related genes

## Abstract

**Objective:**

To probe into the role of pyroptosis-related genes in gastric cancer.

**Methods:**

To establish pyroptosis-related genes, observe their expression in gastric cancer, and analyze the prognosis of pyroptosis-related genes in gastric cancer by single-factor COX, which showed that only GSDME had prognostic significance in gastric cancer. The mRNA expression profiles and lncRNA expression profiles of gastric cancer downloaded from the Cancer Genome Atlas were combined for weighted gene regulatory network analysis, after which the lncRNA nodes of the module to which GSDME belongs were extracted to obtain the lncRNAs−GSDME interactions, which were visualized with Cytoscape network plots. Finally, the effects of GSDME on the proliferation, migration, and apoptosis of gastric cancer cells were observed with CCK8, and flow cytometry.

**Results:**

Our results show that only GSDME has prognostic significance in gastric cancer, and show that it has an important role in a variety of tumors. In addition, our results show that 16 lncRNAs have a significant interaction with GSDME. Finally, the experimental analysis showed that knocking down the expression level of GSDME could affect the growth as well as apoptosis of gastric cancer cells.

**Conclusion:**

The significant prognostic significance of GSDME in gastric cancer and the fact that affecting GSDME expression inhibits gastric cancer cell growth suggest that GSDME can be used as a predictive biomarker.

## Introduction

Stomach cancer has caused many deaths. Gastric cancer causes more than 720,000 deaths per year (1, 2). More than 90% of gastric cancers are adenocarcinomas, which can be subdivided into cardia and non-cardia tumors, respectively, according to the site of the tumor (3). Cardia cancer occurs in the region adjacent to the esophagogastric junction and therefore has the same epidemiological features as esophageal adenocarcinomas. Non-cardia carcinomas, also known as distal gastric carcinomas, often occur in the lower part of the stomach (4). Due to the lack of typical early signs, 40% of them have metastatic disease at the time of diagnosis (5). When patients are found to have metastases, they are already in the middle to late stages of gastric cancer, and Patients with advanced gastric cancer are bound to die, ranging from approximately 10% to 30% at five years (6).

Pyroptosis amplifies or maintains inflammation by releasing pro-inflammatory cellular contents that tightly control the inflammatory response and orchestrate antimicrobial host defenses. It is an innate immune effector mechanism for fighting intracellular pathogens (7, 8). Heat illnesses are linked to many diseases, with different tissue and genetic backgrounds having different effects on cancer. On the one hand, pyroptosis can inhibit tumor development; on the other hand, as pro-inflammatory death, pyroptosis can form a microenvironment suitable for tumor cell growth, thus promoting tumor growth (9, 10). With the progress of research, the role of pyroptosis in tumors has become more prominent, as it may affect all level of carcinogenesis. Therefore, this study investigated the potential impact of genes related to pyrogenesis in gastric cancer.

## Methods

### Data Acquisition

There were 375 gastric cancer tissue samples and 32 corresponding normal tissue samples. We also downloaded gastric cancer mRNA expression profiles and lncRNA expression profiles from the TCGA dataset for subsequent analysis.

### Pyroptosis-Related Gene Acquisition

The human genes with BP_PYROPTOSIS in their annotation results were NLRC4, NLRP1, AIM2, TREM2, GSDMC, GSDMB, NLRP9, NLRP6, ELANE, NAIP, CASP8, CASP4 GZBP1, APIP, GZMB, GZMA, CASP1, DHX9, GSDMA, GSDME, and GSDMD. Thus, the set of functional genes related to focal death was constructed.

### Expression Analysis

Statistical significance was considered achieved when p < 0.05.

### Pan-Cancer Analysis

The correlation between MSI and GSDME expression levels in pan-cancer was analyzed using the R language with the fmsb package. It was considered statistically significant when p < 0.05. **Table 1** displays 33 tumor abbreviations in the TCGA database.

**Table 1:** TCGA database tumor abbreviations.

Abbr	Full name
ACC	Adrenocortical carcinoma
BLCA	Bladder urothelial carcinoma
BRCA	Breast invasive carcinoma
CESC	Cervical squamous cell carcinoma and endocervical adenocarcinoma
CHOL	Cholangiocarcinoma
COAD	Colon adenocarcinoma
COADREAD	Colon adenocarcinoma/Rectum adenocarcinoma/Esophageal carcinoma
DLBC	Lymphoid neoplasm diffuse large B-cell lymphoma
ESCA	Esophageal carcinoma
FPPP	FFPE Pilot Phase II
GBM	Glioblastoma multiforme
GBMLGG	Glioma
HNSC	Head and neck squamous cell carcinoma
KICH	Kidney chromophobe
KIPAN	Pan-kidney cohort (KICH+KIRC+KIRP)
KIRC	Kidney renal clear cell carcinoma
KIRP	Kidney renal papillary cell carcinoma
LAML	Acute myeloid leukemia
LGG	Brain lower grade glioma
LIHC	Liver hepatocellular carcinoma
LUAD	Lung adenocarcinoma
LUSC	Lung squamous cell carcinoma
MESO	Mesothelioma
OV	Ovarian serous cystadenocarcinoma
PAAD	Pancreatic adenocarcinoma
PCPG	Pheochromocytoma and paraganglioma
PRAD	Prostate adenocarcinoma
READ	Rectum adenocarcinoma
SARC	Sarcoma
SKCM	Skin cutaneous melanoma
STAD	Stomach adenocarcinoma
STES	Stomach and esophageal carcinoma
TGCT	Testicular germ cell tumors
THCA	Thyroid carcinoma
THYM	Thymoma
UCEC	Uterine corpus endometrial carcinoma
UCS	Uterine carcinosarcoma
UVM	Uveal melanoma

### GSEA Analysis

To understand the impact of various biological functional gene sets of GSDME in gastric cancer, GSEA was used for enrichment analysis. GSDME was classified into high and low expression parts. Subsequently, GSEA V3.0 software was used to analyze the enrichment results of genes. A nominal P < 0.05 and a false discovery rate (FDR) < 25% were selected as cut-off criteria. We selected the top five-ranked analysis results.

### Weighted Gene Regulatory Network Analysis

A soft threshold for network construction is first selected. Second, the adjacency matrix is transformed into a topology matrix. The genes are clustered based on the topology matrix using the average chain hierarchy clustering method. We set the minimum base number for each gene network module at 30. After determining the gene modules by the dynamic cut method, the feature vectors of each module are calculated in turn. Next, the modules are clustered, and the closer modules are merged into new modules.

### Sample Collection

The gastric cancer tissues and the corresponding adjacent cancer tissues were collected from 5 GC patients, and the liquid nitrogen was preserved. Two cases were male and three cases were female. Sample collection excluded patients with major diseases. The study was informed by the patient and obtained by the Ethics committee of our hospital (ethical batch number: IIT20210716A).

### Cell Source and Culture

Human gastric cancer cell lines (HS-746T, HT-X2557, MKN74, HTX2401C) and human gastric mucosal cells (GES-1, HT-X1964) were got from Shenzhen Haodi Huatuo Biotechnology Co. These cells were cultured with Dulbecco’s modified Eagle medium. Cells were supplemented with 10% fetal bovine serum and penicillin-streptomycin solution at 37°C and 5% CO2. After reaching 80–90% confluence with wall growth, cells were digested with 25% trypsin for passaging.

### Cell Transfection

Cells were transferred to six-well plates at 2 x 10 five cells/well and incubated overnight at 37°C in an incubator. Si-NC and Si-GSDME were transfected with HS-746T and MKN74 cells using a Lipofectamine 2000 kit according to the kit instructions into DMEM containing 10% FBS and incubated for 6 h after transfection.

### Quantitative Real-Time Polymerase Chain Reaction (qRT-PCR) Assay

Grind the gastric cancer tissue to prepare a cell suspension. Total RNA was extracted from cells using the TRIzol Kit (Invitrogen, USA).Then, 1.7 ≤ OD260/OD280 ≤ 2.1 RNA samples could be used for analysis. Subsequently, the total RNA was reverse transcribed using a reverse transcription kit. The volume was adjusted by 20 μL of total reaction volume and 10 μL of SYBR Premix Ex Taq II (2X), 2 μL of cDNA, 0.8 μL of upstream and downstream primers, and the addition of sterile purified water. The amplification conditions were: 95°C for 30 s, 95°C for 5 s, 60°C for 30 s, and 40 cycles. GAPDH was used as an internal reference for GSDME. All primers were purchased from Shanghai Gene Chemistry Co. The forward primer for GSDME was 5′-TGCCTACGGTGTCATTGAGTT-3′; its reverse primer was TCTGGCATGTCTATGAATGCAAA-3′. The forward primer for GAPDH was 5′-GAGTCAACGGATTTGGTCGT-3′, and its reverse primer was 5′-GACAAGCTTCCCGTTCTCAG-3′. Now, 2–^ΔΔ^Ct was used to analyze the data, and the experiment was repeated three times.

### Cell Counting Kit-8 (CCK8) Assay

Cell proliferation assays were performed using CCK-8 (Beyotime Biotechnology, China) according to the kit’s instructions: Cells were transfected for 24 h and incubated in 96-well plates at 2.5×10 ^3^ cells/well. After 24, 48, and 72 h of incubation, 10 μL of CCK-8 solution was added to each well and incubated at room temperature for 2 h. Subsequently, the optical density of each well at 450 nm was detected using an enzyme marker. The experiment was repeated three times.

### Flow Cytometry

The Annexin V-FITC Apoptosis Detection Kit (Beyotime, China) measured the apoptosis in gastric cancer cells treated as indicated. The experiments were repeated three times.

### Statistical Analysis

The data obtained were plotted and analyzed using GraphPad 8. Comparisons between groups were made using independent t-tests, and comparisons between multiple parts were made using one-way ANOVA.

## Results

### Expression Levels of Pyroptosis-Related Genes in Gastric Cancer

**Figure 1:**
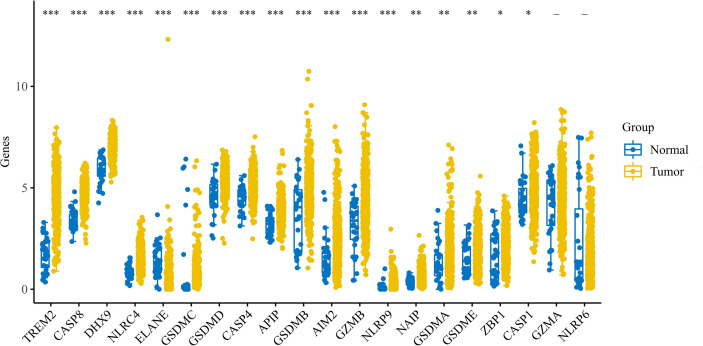
Expression of pyroptosis-related genes in gastric cancer. Expression of pyroptosis-related genes in gastric cancer. * indicates p < 0.05 for comparison between gastric cancer and paraneoplasia; ** indicates p < 0.01 for comparison between gastric cancer and paraneoplasia; *** indicates p < 0.001 for comparison between gastric cancer and paraneoplasia.

We performed bulk expression analysis obtained and observed the expression of pyroptosis-related genes in gastric cancer (n = 375) and paracancerous tissue (n = 32). Most of the pyroptosis-related genes were upregulated in gastric cancer. See **Figure 1**.

### Prognostic Analysis of Pyroptosis-Related Genes

**Figure 2:**
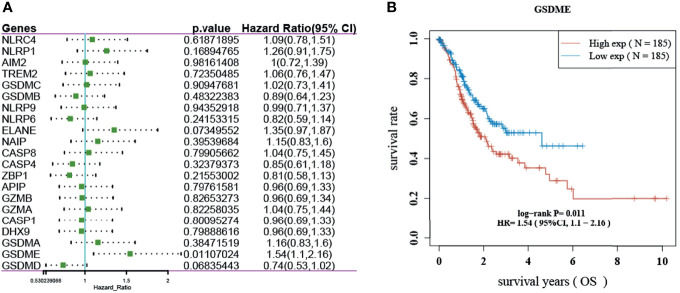
Prognosis of pyroptosis-related genes in gastric cancer. **(A)** Forest plot showing the OS prognosis of pyroptosis-related genes in gastric cancer. **(B)** KM survival curve of GSDME in gastric cancer.

The results showed that only GSDME had prognostic significance in gastric cancer. The KM survival curve analysis also showed that the high-level survival of GSDME was lower than the low-level survival of GSDME. See **Figure 2**.

### Role of GSDME in Multiple Tumors

**Figure 3:**
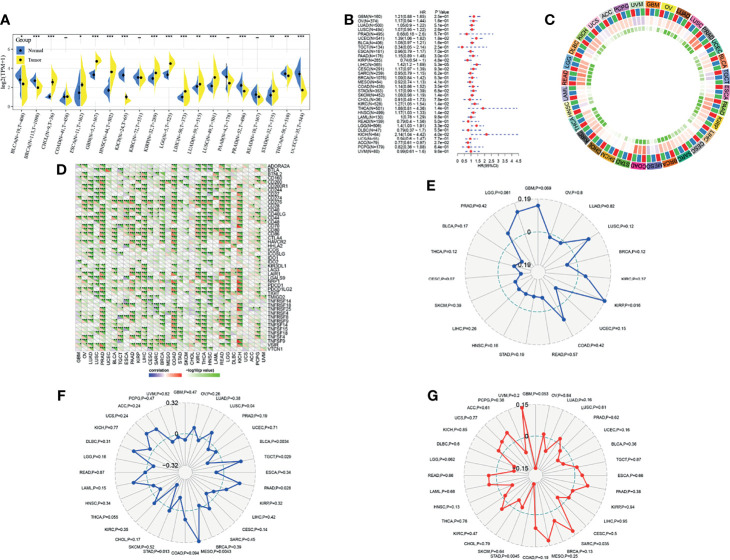
Analysis of GSDME in multiple tumors. **(A)** Expression of GSDME in multiple tumors (* indicates p < 0.05 for comparison between cancer and paracancer; ** indicates p < 0.01 for comparison between cancer and paracancer; *** indicates p < 0.001 for comparison between cancer and paracancer). **(B)** Prognostic analysis of GSDME in multiple tumors. **(C)** Correlation of GSDME with immune microenvironment scores in multiple tumors (blue, minimum color; red, maximum value color; green, minimum p-value color). **(D)** Correlation of GSDME with immune checkpoints in multiple tumors. **(E)** Correlation of GSDME with immune neoantigens in multiple tumors. **(F)** Correlation of GSDME with immune mutational load in multiple tumors. **(G)** correlation of GSDME with microsatellites in multiple tumors.

To continue to probe the role of GSDME in tumors, we also observed the expression of GSDME in multiple tumors. GSDME was highly expressed in CHOL, ESCA, GBM and so on, and low expressed in BLCA, BRCA, KICH, PRAD, THCA, and UCEC. A univariate cox analysis revealed the prognostic significance of GSDME in BLCA, KIRP, LIHC, KIRC, HNSC, LGG, KICH, and ACC. An immune checkpoint correlation analysis demonstrated that GSDME was closely associated with immune checkpoints TNFSF9, TNFSF15, TNFSF18, TNFSF4, TNFRSF25, TNFRSF4, TNFRSF8, LGALS9, NRP1, CD276, CD40, and CD200 in gastric cancer. A neoantigen analysis depicted that GSDME was only correlated in KIRP. An immunomutational load correlation indicated that GSDME was significantly correlated with immunomutational load in STAD, MESO, PAAD, TGCT, BLCA, and LUSC. Microsatellite correlation analysis showed that GSDME was significantly correlated with microsatellite instability in STAD and SARC. See **Figure 3**.

### GSEA Analysis

**Figure 4:**
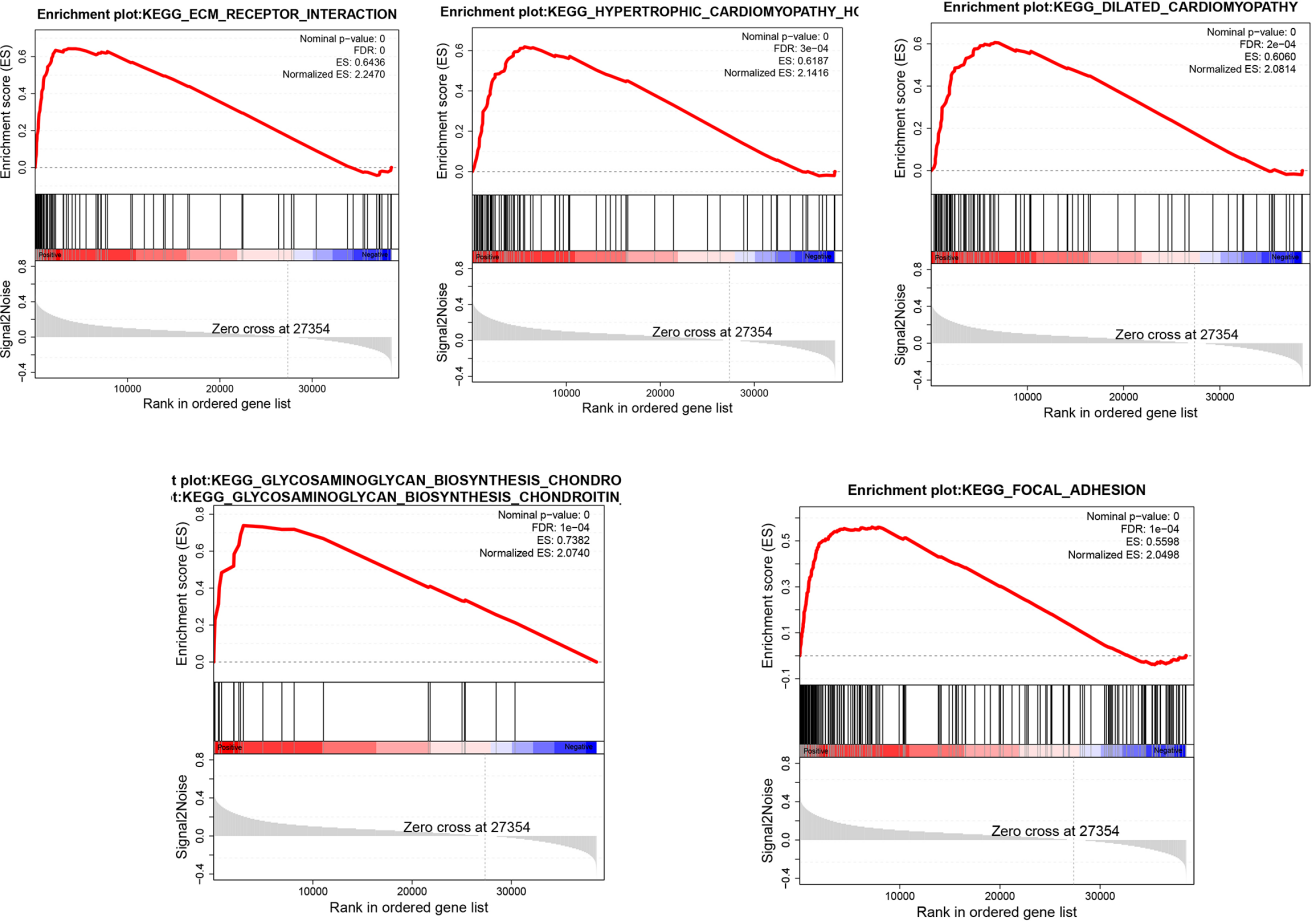
Biological processes involved in the high expression of GSDME in gastric cancer.

We divided the samples into 2parts according to the median expression levels of GSDME in gastric cancer and then analyzed the biological processes or signaling pathways involved in high GSDME expression in gastric cancer. We only screened the top five-ranked signaling pathways. The analysis revealed that the high expression of GSDME in gastric cancer was found in ECM_RECEPTOR_INTERACTION, HYPERTROPHIC_CARDIOMYOPATHY_ HCM, DILATED_CARDIOMYOPATHY, GLYCOSAMINOGLYCAN_ BIOSYNTHESIS_CHONDROITIN_SULFATE, and FOCAL_ADHESION, which have important roles. See **Figure 4**.

### GSDME and the Stemness Score of Gastric Cancer Cells

**Figure 5:**
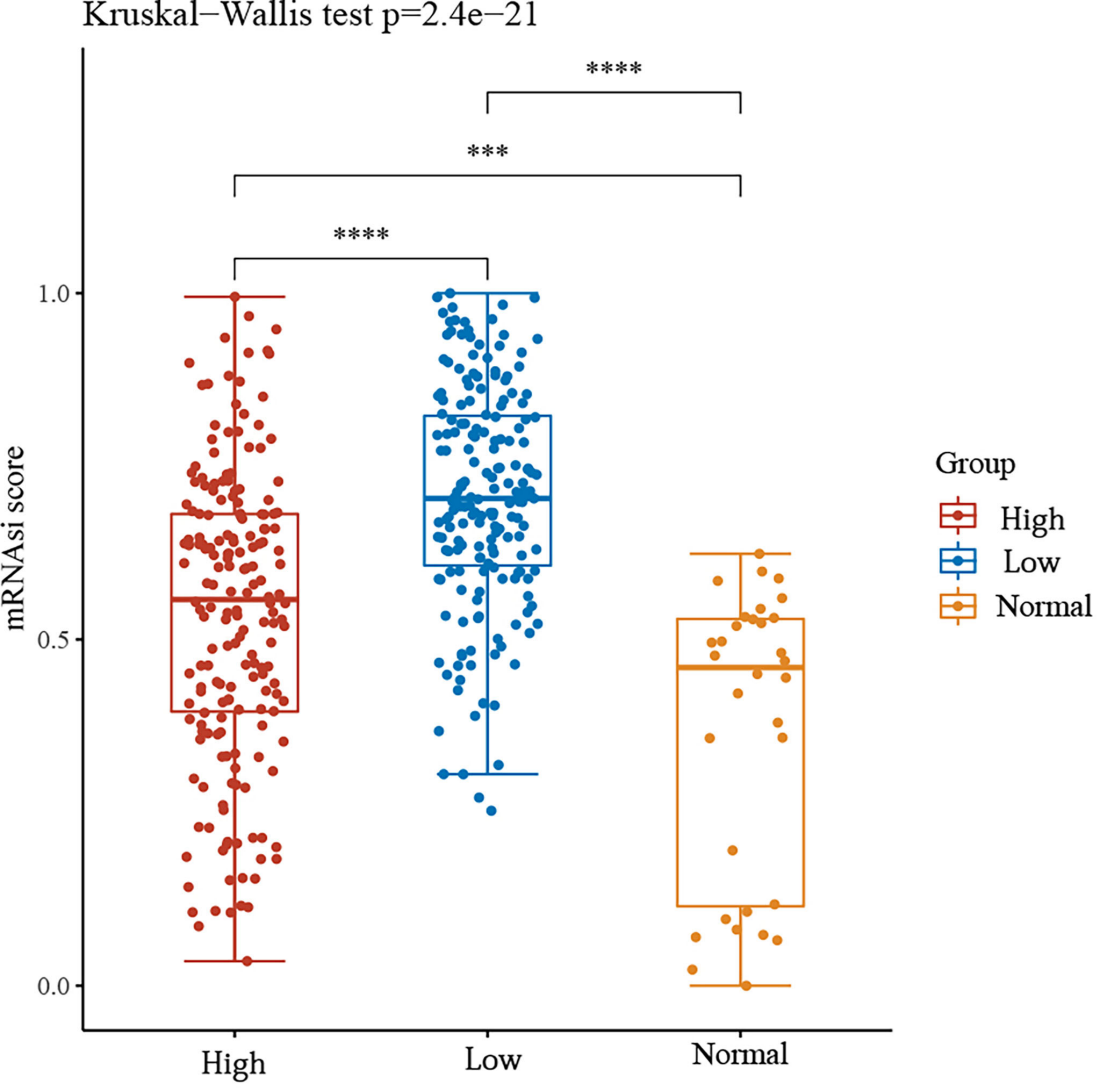
mRNAsi scores between high and low GSDME expression groups and normal groups. ***P < 0.001, ****P < 0.0001.

We then divided the group into high and low expression groups based on the median GSDME expression. The mRNAsi scores between the two groups and the normal group were observed. The results showed that the mRNAsi score was significantly higher in the GSDME high expression group compared to the normal group. Surprisingly, the mRNAsi score was again significantly higher in the GSDME low expression group compared to the high expression. This may be a shift in the progression of gastric cancer, but the exact reason for this remains to be explored. See **Figure 5**.

### WGCNA Analysis

**Figure 6:**
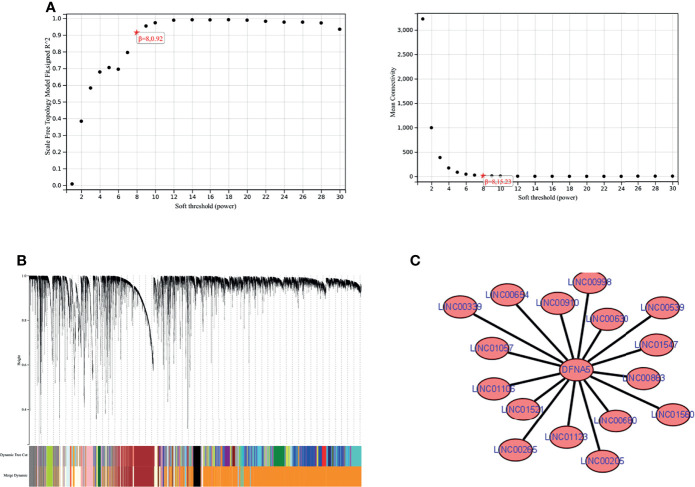
WGCNA analysis. **(A)** soft threshold determination. **(B)** gene clustering map. **(C)** lncRNAs-GSDME interaction network map.

We combined the mRNA expression profiles obtained from the TCGA database with lncRNA expression profiles and performed WGCNA analysis. We found the module darkorange2 to which GSDME belongs. Next, we extracted the lncRNA nodes belonging to this module and obtained 16 lncRNAs with interactions with GSDME, namely, LINC01106, LINC01547, LINC00265, LINC00910, LINC01560, LINC01123, LINC00654, LINC01521, LINC00998, LINC00680, LINC00339, LINC00630, LINC00205, LINC01057, LINC00539, and LINC00863. Finally, we used Cytoscape (https://cytoscape.org/) for visualization. See **Figure 6**.

### Experimental Observation of the Effect of GSDME on Gastric Cancer Cells

**Figure 7:**
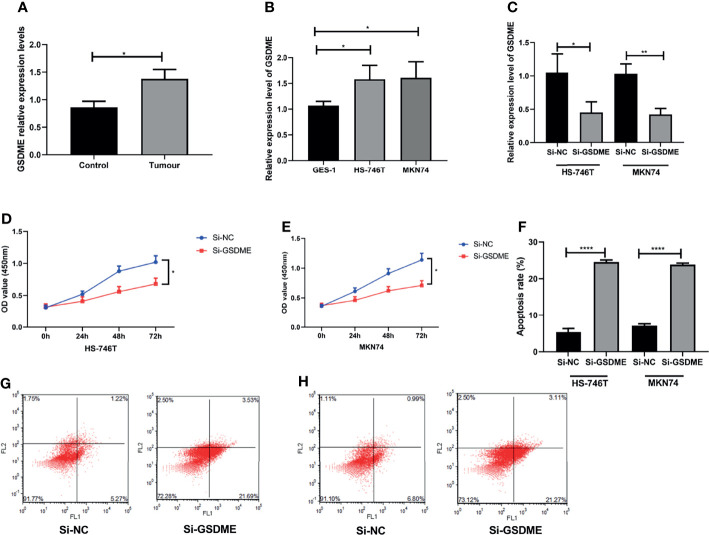
Effect of the knockdown of GSDME expression on gastric cancer cells. **(A)**. Relative expression levels of GSDME in gastric cancer and adjacent to paracancerous tissues. **(B)** Expression of GSDME in human gastric cancer cell lines HS-746T and MKN74 and gastric mucosal cells GES-1. **(C)** Transfection efficiency. **(D)** Effect of reduced GSDME levels on the proliferation of HS-746T cells. **(E)** Effect of reduced GSDME levels on the proliferation of MKN74 cells. **(F)** Promotion of apoptosis after GSDME levels were reduced. **(G)** Flow apoptosis diagram based on HS-746T cells. **(H)** Flow apoptosis diagram based on MKN74 cells. *P < 0.05, **P < 0.01, ****P < 0.0001.

Finally, we performed experiments to observe whether GSDME influences the biological behavior of gastric cancer cells. We first observed the expression of GSDME in gastric cancer tissue, gastric cancer cell lines HS-746T and MKN74 and gastric mucosal cells GES-1. The expression level of GSDME was significantly higher in gastric cancer tissue, HS-746T and MKN74 than in GES-1. We then further observed the effect of GSDME on gastric cancer cells by knocking down the expression level of GSDME to observe its effect on gastric cancer cells. The expression level of GSDME was reduced, the growth ability of gastric cancer cells were significantly slowed, and the apoptosis of cells was markedly increased. See **Figure 7**.

## Discussion

GSDME related to the induction of secondary necrosis/scarring death. GSDME expression may also control the transition, such that in response to apoptotic stimuli, cells lacking GSDME expression undergo apoptosis without progressing. GSDME also plays distinct roles in a variety of tumors. In the present study, the expression of GSDME also differed in assorted tumors. Moreover, GSDME was closely associated with immune checkpoints in most tumors, including gastric cancer. GSDME expression enhanced the phagocytosis of tumor cells by tumor-associated macrophages and enhanced the number and function of tumor-infiltrating natural killer cells and CD8+ T cells, It can be cleaved by activated caspase-3 to produce its N-terminal fragment (GSDME-NT) (11, 12). It has also been indicated that targeted drugs induce GSDME-mediated cellular scorching in melanoma, linking the tumor immune microenvironment to T cell-mediated anti-tumor immunity (13). It is suggested that GSDME may related to cancer therapy and antitumor immunity, but the potential effect of GSDME on immune cells in gastric cancer needs to be investigated in more depth.

In rectal and esophageal squamous cell carcinoma also significantly higher than normal tissue and promote tumor progression (14, 15). In the present study, only GSDME among several pyroptosis genes had prognostic significance in gastric cancer. The KM curve revealed that the high expression of GSDME was associated with a poor prognosis for patients. The stemness score also showed a significantly higher mRNAsi score in the GSDME high expression group compared to the normal group. Interestingly, the mRNAsi score was again significantly higher in the GSDME low expression group compared to the high expression. However, the exact mechanism of development remains to be investigated.

Tumor development is inseparable from the proliferation and migration of cancer cells. Conversely, the inhibition of cancer cell proliferation may stunt tumor growth. In breast cancer, cell survival is strongly correlated with GSDMB expression. In addition, reports have demonstrated that GSDMB overexpression reduces cell viability (16). Gene deletion of GSDME promoted drug resistance (17). Another study reported that the knockdown of GSDME shifted loplatin-induced cell death from cell scorching to apoptosis, but did not affect the growth and tumor formation of colon cancer cells treated with loplatin (18). Although GSDME has been less studied in gastric cancer, one study has shown that GSDME converts chemotherapy-induced caspase-3-dependent apoptosis to cellular scorching in gastric cancer cells (19).Our *in vitro* experiments also show that changes in GSDME expression may affect the biological behavior of gastric cancer cells, which may provide new ideas for the treatment of gastric cancer.

LncRNA is a transcript with more than 200 nucleotides and no protein-coding potential. However, it is frequently dysregulated in cancer (20, 21). In therapeutic agents, LncRNAs can exert clinical therapeutic effects on tumors by inhibiting the transcription of mRNAs and blocking their function in combination with proteins (22, 23). In the present study, we found that 16 lncRNAs had reciprocal relationships with GSDME by WGCNA analysis, namely, LINC01106, LINC01547, LINC00265, LINC00910, LINC01560, LINC01123, LINC00654, LINC01521 LINC00998, LINC00680, LINC00339, LINC00630, LINC00205, LINC01057, LINC00539, and LINC00863. However, no clinical studies indicate that these lncRNAs have a regulatory relationship with GSDME. Correspondingly, more in-depth studies are needed.

In conclusion, GSDME is highly expressed in gastric cancer. The knockdown of GSDME expression can inhibit the growth of gastric cancer cells. GSDME, then, has prognostic significance in gastric cancer and can be used as a predictive biomarker.

## Data Availability Statement

The original contributions presented in the study are included in the article/supplementary material. Further inquiries can be directed to the corresponding authors.

## Author Contributions

These authors contributed equally: JY and GC. All authors contributed to the article and approved the submitted version.

## Funding

The Key Research and Development Project of Zhejiang Province (2019C03071).

## Conflict of Interest

The authors declare that the research was conducted in the absence of any commercial or financial relationships that could be construed as a potential conflict of interest.

## Publisher’s Note

All claims expressed in this article are solely those of the authors and do not necessarily represent those of their affiliated organizations, or those of the publisher, the editors and the reviewers. Any product that may be evaluated in this article, or claim that may be made by its manufacturer, is not guaranteed or endorsed by the publisher.
